# Exploring the role of melatonin in managing sleep and motor symptoms in Parkinson’s disease: a pooled analysis of double-blinded randomized controlled trials

**DOI:** 10.1007/s10072-025-08221-8

**Published:** 2025-05-19

**Authors:** Ahmed Samy Badran, Hamza Khelifa, Mohamed Ibrahim Gbreel

**Affiliations:** 1 Faculty of Medicine, Ain Shams University, Cairo Governate, Cairo, 1181 Egypt; 2 Faculty of Medicine, University of Oran 1 Ahmed Ben Bella, Oran, Algeria; 3 Faculty of Medicine, October 6 University, Giza, Egypt; 4 Department of Cardiology, Egyptian railway Medical Educational Centre, Cairo, Egypt

**Keywords:** Melatonin, Non-Motor symptoms, Parkinson’s disease, Sleep disorders, Meta-analysis

## Abstract

**Background:**

Parkinson’s disease (PD), a progressive neurodegenerative disorder, often involves sleep disturbances, affecting 88–98% of patients. Melatonin, a sleep-regulating neurohormone, shows the potential to improve sleep quality and non-motor symptoms in PD.

**Aim:**

To evaluate melatonin’s efficacy and safety in PD patients with sleep disorders.

**Methods:**

We systematically searched PubMed, Scopus, Web of Science, and Cochrane till January 2025. The risk of bias in the included studies was evaluated using the Cochrane risk-of-bias tool. Dichotomous outcomes were expressed as risk ratios (RRs) with 95% confidence intervals (CIs), while continuous outcomes were reported as mean differences (MDs) with 95% CIs.

**Results:**

We retrieved 2537 records. Five double-blinded RCTs were finally included. The meta-analysis revealed a significant improvement in sleep quality, as measured by the Pittsburgh Sleep Quality Index (PSQI), in the melatonin group compared to placebo (MD= -1.88, 95% CI: [-3.07, -0.68], *P* = 0.002). However, no significant differences were observed for the Epworth Sleepiness Scale (MD= -1.04 CI: [-2.81, 0.73], *P* = 0.25), total sleep time (MD = 14.85 min CI: [-5.45, 35.16], *P* = 0.15), sleep efficiency, sleep latency, REM sleep latency, frequency of arousals, or REM Sleep Behavior Disorder Screening Questionnaire (RBDSQ; MD = 0.74, *P* = 0.50). For Parkinson’s disease-related outcomes, melatonin significantly improved Non-Motor Symptom Scale (NMSS) scores but showed no significant effects on UPDRS Part III scores or Parkinson’s Disease Quality of Life.

**Conclusion:**

Melatonin improves subjective sleep quality and non-motor symptoms in PD patients with a favorable safety profile, but effects on objective measures and motor symptoms remain inconclusive.

**Trial registry number:**

This meta-analysis was registered on PROSPERO. Registration Number: CRD42024619496.

**Supplementary Information:**

The online version contains supplementary material available at 10.1007/s10072-025-08221-8.

## Introduction

Parkinson’s disease (PD), a prevalent neurodegenerative disorder [1], is projected to affect approximately 13 million people worldwide by 2040, creating a significant burden on society [2]. PD predominantly affects the central nervous system, impacting both motor and non-motor systems. PD’s Symptoms progress over time, with non-motor complications becoming more prominent in later stages. Prevalent motor symptoms encompass tremors, bradykinesia, rigidity, and balance impairments, generally referred to as parkinsonism [3]. The risk of falls, dementia, and neuropsychiatric issues—such as sleep abnormalities, the focus of this study—increases in advanced stages [4].

Sleep abnormalities are common in patients with PD, affecting 88–98% of patients. It impacts the individual’s health and quality of life, making it difficult for them to recover and complicating the management of the case. These abnormalities include daytime sleepiness with sleep attacks, rapid eye movement sleep behavior disorder (RBD), restless leg syndrome, and insomnia [5].

Multiple factors are thought to cause sleep disturbance in PD patients, including the physiopathology of the disease itself, the medication side effects, and the patient’s emotional status, as depression and anxiety [6].

Melatonin, a pineal gland neurohormone, has an essential role in sleep promotion and the regulation of sleep/wake cycles [7]. Its secretion rhythm is blunted and significantly reduced in PD, contributing to excessive daytime sleepiness [8]. Several trials highlighted its potential role in regulating sleep disturbance by improving overall sleep quality and reducing the time it takes to fall asleep [9]. Additionally, studies suggest that melatonin supplementation may help alleviate some of the motor and non-motor symptoms associated with PD [10].

Beyond its promising role in improving sleep quality, melatonin may act as a neuroprotective agent by modulating neuroinflammation [11] and has been shown to reduce oxidative stress and neuroinflammatory markers associated with PD [12, 13]. It decreases the activity of Cyclooxygenase 2 (COX-2) activity and serum concentration of nitric oxide metabolites, and lipo-peroxides [14].

Some trials reported no effect of melatonin on RBD in PD [15, 16], while others observed a minor, clinically insignificant improvement in total sleep time [17].

Therefore, we conducted a systematic review and meta-analysis to evaluate the efficacy and safety of melatonin in patients with Parkinson’s disease and sleep disorders and explore its usage and impact on multiple scores and outcomes.

## Materials and methods

The methodology of this study is based on the Cochrane Handbook of Systematic Reviews on interventions [18]. During the process of drafting our manuscript, we followed the recommended reporting items for the Preferred Reporting Items for Systematic Reviews and Meta-Analyses (PRISMA) statement guidelines [19]. Our protocol was prospectively registered on PROSPERO with ID: (CRD42024619496).

### Search strategy

We conducted a comprehensive systematic search across four databases—PubMed (MEDLINE), Scopus, Cochrane Library, and Web of Science—using specific keywords and Medical Subject Headings (MeSH) terms related to the scope of this study, namely “Melatonin” and “Parkinson’s disease,” up to January 2025. Additionally, two independent authors (A.S.B. AND H.K.) reviewed the reference lists of relevant articles to identify potential studies. The strategy details and each database search are demonstrated in Supplementary Material 1.

### Eligibility criteria

In this review, we applied the following Population, Intervention, Comparator, Outcomes, and Study design (PICOS) criteria: (P) patients with Parkinson’s disease, (I) any form of melatonin therapy, (C) placebo, (O) efficacy and safety, and (S) only randomized controlled trials (RCTs) were eligible for inclusion. We excluded studies that did not specifically focus on PD. Additionally, we excluded crossover studies, case reports, case series, conference abstracts, letters to the editor, opinion papers, review articles, non-randomized clinical trials, editorials, animal studies, notes, posters, theses, books, and conference proceedings.

### Screening and study selection

We collected the different records from the various databases and removed duplicates using EndNote software [20]. The initial search results were imported into the Rayyan software [21]. The screening was done in two steps: title and abstract screening, followed by full-text screening for eligibility criteria. Two independent authors (A.S.B. AND H.K.) performed each step, then the findings were compared. Another author (M.I.G.) was included in the discussion to achieve consensus in conflicts.

### Risk of bias assessment and certainty of evidence

The included RCTs were assessed for bias quality using the revised version of the Cochrane Collaboration tool (R.o.b.2) [22]. It covers the following domains: (1) randomization process, (2) Deviations from intended interventions, (3) Missing outcome data, (4) Measurement of the outcome, (5) Selection of the reported result, and (6) other sources of bias. Also, we assessed the certainty of evidence through the Grading of Recommendations, Assessment, Development, and Evaluation (GRADE) approach, in which the quality of evidence is classified into four levels: high, moderate, low, and very low certainty. Each outcome is assessed using five domains: risk of bias, inconsistency, indirectness, imprecision, and publication bias [23, 24].

### Data extraction

Two independent authors (A.S.B. AND H.K.) manually extracted data from the included studies using spreadsheets, capturing key baseline and outcome information. A third author (M.I.G.) was included to achieve consensus in conflicts. The extracted data included the study ID, year of conduct, country, study design and phase, protocol NCT number, and inclusion criteria. Additionally, we recorded details on follow-up duration and the total number of participants. For both the intervention and comparator groups, we documented the name, dose, any add-on therapy, route of administration, baseline participant characteristics (e.g., age, sex, race distribution, and duration of diagnosis at baseline), Parkinson’s Disease Questionnaire-39 (PDQ-39) scores, Movement Disorder Society-Unified Parkinson’s Disease Rating Scale (MDS-UPDRS) scores (Parts I–IV), and Parkinson’s disease medication usage.

### Endpoints

The primary endpoint of this systematic review and meta-analysis was the change in sleep quality, as measured by the Pittsburgh Sleep Quality Index (PSQI). The PSQI was selected due to its validated and comprehensive assessment of sleep quality. Secondary endpoints included additional sleep-related outcomes, such as the Epworth Sleepiness Scale (ESS) for daytime sleepiness, total sleep time (minutes), sleep efficiency (%), sleep latency (minutes), REM sleep latency (minutes), frequency of arousals, and the REM Sleep Behavior Disorder Screening Questionnaire (RBDSQ) score. These outcomes were chosen to provide more evaluation of the various dimensions of sleep architecture and disturbances. Parkinson’s disease-related outcomes, including the UPDRS Part III scores for motor symptoms, the PDQ-39 scale, the Non-Motor Symptom Scale (NMSS), and Adverse events, were also assessed to explore melatonin’s broader effects on PD symptomatology.

### Statistical analysis

We used the Review Manager software (RevMan 5.4) [Computer program] from the Cochrane Collaboration. If the continuous variables’ mean or standard deviations (SDs) were not reported, we used validated conversion methods to estimate the mean and SD from available data (e.g., medians, interquartile ranges (IQR), or ranges) [25, 26]. Continuous data were analyzed as mean difference (MD) and 95% confidence interval CI, while dichotomous data as risk ratio (RR) and 95% (CI). Statistical heterogeneity among the studies was assessed by visual inspection of the forest plot, besides using I-squared (I^2^) and chi-squared (Chi2) statistics. I^2^ values of 50% were indicative of high heterogeneity [27]. We adopted a leave-one-out sensitivity analysis in case of high heterogeneity. Given that funnel plots and Egger’s test are unreliable for fewer than ten pooled studies [28], we couldn’t assess publication bias.

## Results

### Literature search

**Fig. 1:**
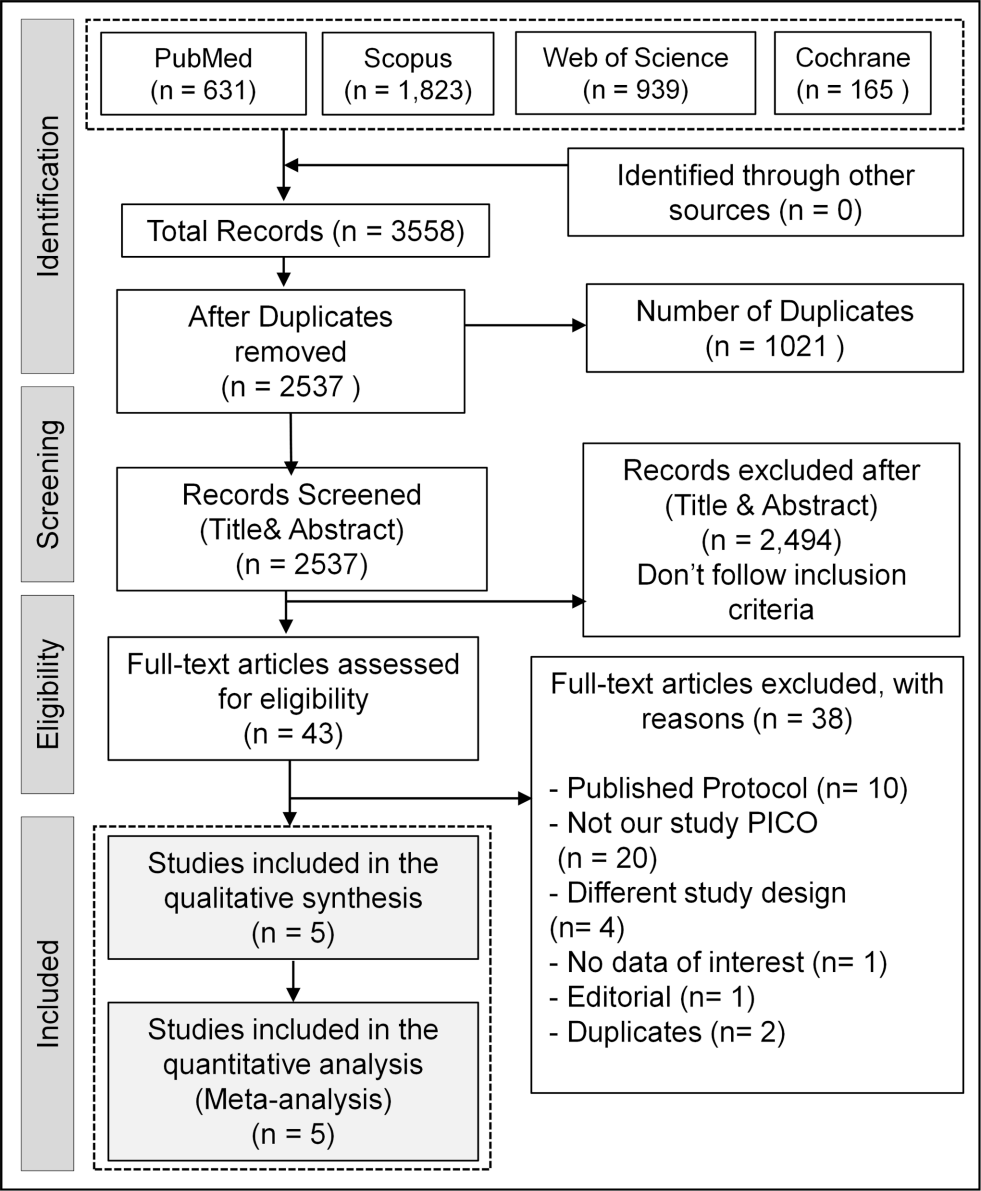
PRISMA flow diagram of the literature search

After searching databases, we retrieved 2537 records. According to the declared selection criteria, five double-blind RCTs [15, 16, 29–31] were finally pooled in our study, with 219 patients diagnosed with Parkinson’s disease. Figure 1 shows the processes of literature identification and study selection.

### Characteristics of the included studies

Five RCTs [15, 16, 29–31] published between 2007 and 2024 were included, comprising a total of 219 patients with a range of mean ages from 58.4 to 67.9 years old. The follow-up duration ranged from 4 to 16 weeks. These studies compared the effects of oral melatonin intake against placebo control groups. Tables 1 and 2 present the characteristics of the included studies and participants, along with detailed summaries.

**Table 1:** Summary of the included studies

Study ID	Design	Protocol no.	Setting	Sample Size	Study Duration (Year)	Follow up duration	Population Definition	Primary Outcomes
**Ahn et al. 2019**	RCT, double-blind	NCT 03258294.	Korea	34	January 2016 to July 2018	4 weeks	Patients aged 55–80 years with a PSQI score greater than 5 were included in the study	(PSQI) Pittsburgh Sleep Quality Index
**Kakhaki et al. 2020**	RCT, double-blind	IRCT20170513033941N29	Iran	51	January 2018 to February 2018	12 weeks	Individuals aged 50–90 years diagnosed with Parkinson’s Disease (PD) based on the UK PD Society Brain Bank criteria, along with rapid eye movement behavior disorder (RBD) or restless leg syndrome	High-sensitivity C-reactive protein and UPDRS score
**Gilat et al. 2020**	RCT, double-blind	ACTRN12613000648729	Australia	30	NA	16 weeks	Eligible participants were over 18 years old, had a Mini-Mental State Examination (MMSE) score of 24 or higher or met ICSD-2 criteria for RBD, scored 5/13 or higher on the RBD Screening Questionnaire (RBDSQ), experienced at least two RBD events involving dream enactment or sleep-related injuries in the 4 weeks prior to randomization, and maintained stable medications for 1 month before randomization	RBD (REM Sleep Behavior Disorder) events
**Sugumaran et al. 2024**	RCT, double-blind	CTRI/2023/08/056190	India	86	August 2023 to February 2024	8 weeks	Participants aged 30 years or older with sleep problems, defined by PSQI score greater than 5	Sleep quality: Measured using the PSQI. Daytime somnolence: Measured using the Epworth Sleepiness Scale (ESS).
**Medeiros et al. 2007**	RCT, double-blind	NA	Brazil	18	NA	4 weeks	Patients with Hoehn and Yahr stage I to III Parkinson’s Disease, aged 41–68 years at onset, disease duration of 2–23 years, and showing therapeutic response to levodopa while maintaining stable antiparkinsonian medication for 30 days	Subjective sleep quality: Evaluated using the PSQI Objective sleep quality: Evaluated using Polysomnography (PSG)

**NA**: Not available; **RCT**: Randomized controlled trial; **PD**: Parkinson’s disease; **PSQI**: Pittsburgh Sleep Quality Index

**Table 2:** Baseline characteristics of the included studies

Study ID	Arm	Sample	Age (Year), Mean ± SD	Gender, Male (%)	BMI (Kg/m2), Mean ± SD	PD History										
						Time since diagnosis (years), mean ± SD	H&Y stage, mean ± SD	PDQ-39, Mean ± SD	MDS-UPDRS scores				PD medication — no. (%)			
									**Part I**	**Part II**	**Part III**	**Part IV**	**Levodopa**	**MAO-B inhibitor**	**Dopamine agonist**	**Untreated**
Sugumaran et al. 2024	Melatonin	35	60.3 ± 6.3	22 (62.9%)	NR	3.0 ± 1.8	1.8 ± 0.6	60.0 ± 12.4	NR	NR	57.6 ± 10.6	NR	35 (100%)	2 (2.33%)	13 (15%)	0 (0)
	Placebo	38	58.4 ± 8.8	23 (60.5%)	NR	3.6 ± 3.6	1.8 ± 0.5	61.2 ± 8.9	NR	NR	56.8 ± 9.1	NR	38 (100%)			
Kakhaki et al. 2020	Melatonin	25	64.4 ± 8.2	16 (64.0%)	25.1 ± 2.1	5.7 ± 1.9	NR	NR	17.1 ± 7.2	20.9 ± 8.3	31.7 ± 14.1	3.7 ± 2.8	51 (100%)	NR	NR	NR
	Placebo	26	66.3 ± 9.3	16 (61.5%)	25.1 ± 3.0	5.5 ± 2.1	NR	NR	14.0 ± 4.3	20.7 ± 8.1	30.1 ± 10.9	5.3 ± 2.5		NR	NR	NR
Gilat et al. 2020	Melatonin	15	65.3 ± 6.9	12 (80%)	NR	5.07 ± 3.9	2 (0-2.5) *	32 (20.8–66.3) *	11.1 ± 5.0	11.0 ± 6.9	27.3 ± 19	1.40 ± 1.6	2 (13.3%)	1 (6.7%)	0 (0)	1 (6.7%)
	Placebo	15	67.9 ± 5.3	13 (86.7%)	NR	6.13 ± 4.4	2 (1-2.5) *	27 (19.0 − 50.0) *	11.9 ± 6.9	10.3 ± 7.2	29.9 ± 16	2.80 ± 4.1	5 (33.3%)	0 (0)	1 (6.7%)	0 (0)
Ahn et al. 2019	PRM	16	66.0 ± 7.5	8 (50%)	25.0 ± 2.7	5.0 ± 5.9	1.7 ± 0.5	33.5 ± 19.2	NR	NR	15.2 ± 5.9	NR	NR	NR	NR	NR
	Placebo	18	64.6 ± 6.5	9 (50%)	23.1 ± 2.3	4.2 ± 4.4	1.7 ± 0.8	31.3 ± 24.1	NR	NR	15.2 ± 10.8	NR	NR	NR	NR	NR
Medeiros et al. 2007	Melatonina	8	62.90 ± 7.78	7 (87.5%)	NR	6.40 ± 2.59	NR	NR	NR	13.5 ± 7.94	16.6 ± 6.9	5.2 ± 4.8	18 (100%)	NR	NR	NR
	Placebo	10	60.70 ± 6.65	7 (70%)	NR	7.70 ± 6.52	NR	NR	NR	15.3 ± 8.7	16.3 ± 8.7	6.0 ± 4.4		NR	NR	NR

* Data are presented in median & IQR

**PD**: Parkinson’s Disease, **(H&Y)**: Hoehn and Yahr stages of Parkinson’s disease, **PDQ-39**: The Parkinson’s Disease Questionnaire 39, **NR**: Not reported

### Risk of bias

**Fig. 2:**
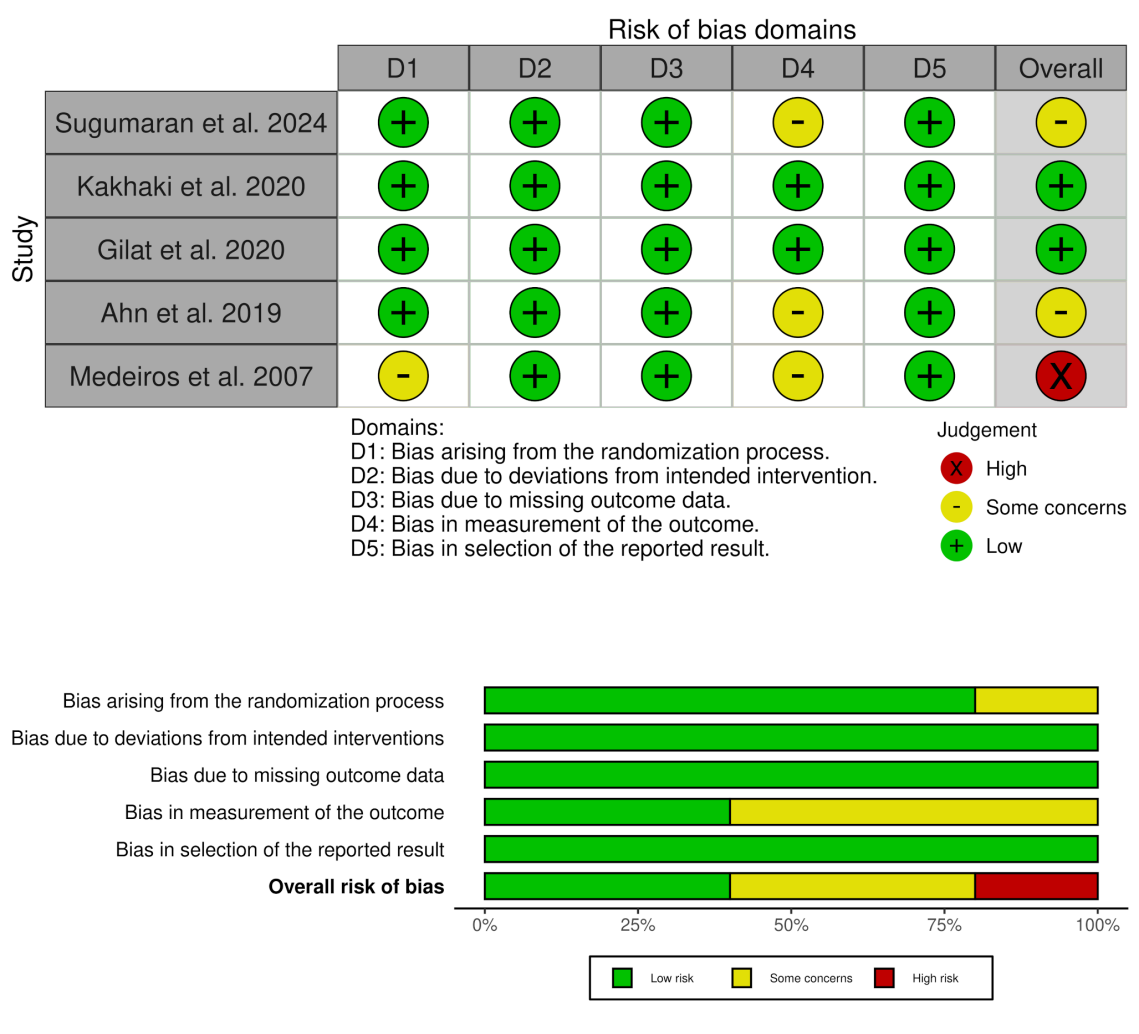
Risk of bias assessment of the included studies

Using the Cochrane Risk of Bias 2 (R.o.b. 2) tool, most studies demonstrated a generally low risk of bias, with some concerns primarily related to measurement bias. Notably, Medeiros et al. (2007) had a high overall risk of bias due to issues with randomization and outcome measurement [30]. A detailed summary of the risk of bias assessment across domains for each study is presented in Fig. 2.

### Outcomes

#### Sleep-related outcomes

##### Pittsburgh sleep quality index (PSQI)

**Fig. 3:**
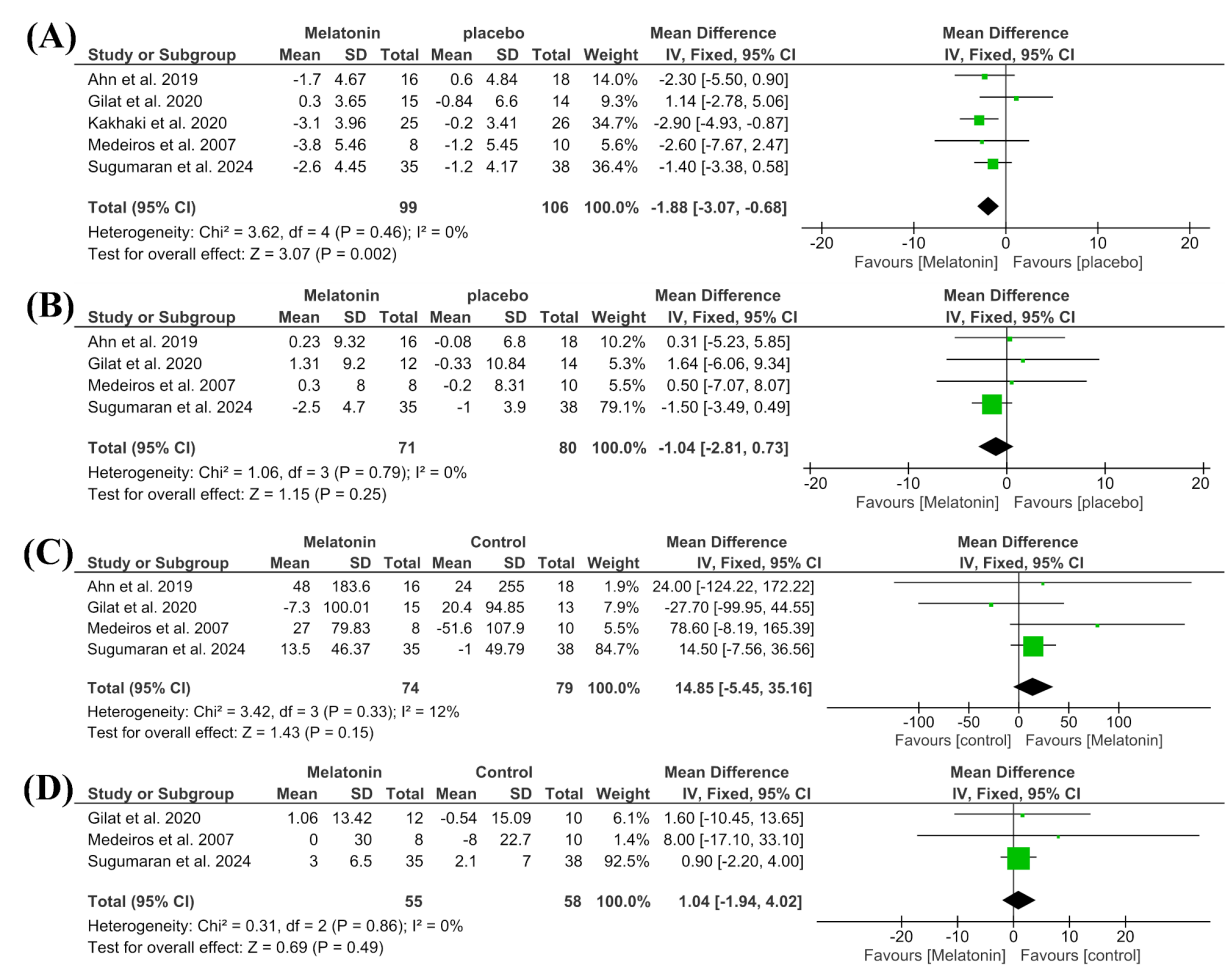
Forest plots of sleep-related outcomes, including (**A**) Pittsburgh Sleep Quality Index (PSQI), (**B**) Epworth Sleepiness Scale (ESS), (**C**) Total Sleep Time (minutes), and (**D**) Sleep Efficiency (%)

The pooled meta-analysis of the included studies demonstrated a significant difference in the PSQI between the melatonin and placebo groups, favoring the melatonin group (MD= -1.88, CI: [-3.07, -0.68], *P* = 0.002). There is no significant heterogeneity between the included studies (*P* = 0.46, I² = 0%) (Fig. 3A).

##### Epworth sleepiness scale (ESS)

The meta-analysis for ESS showed no significant difference between the melatonin and placebo groups (MD = -1.04, CI: [-2.81, 0.73], *P* = 0.25) with no significant heterogeneity (*P* = 0.79, I² = 0%) (Fig. 3B).

##### Total sleep time (minutes)

The meta-analysis for total sleep time showed no significant difference between both groups (MD = 14.85, CI: [-5.45, 35.16], *P* = 0.15) with low heterogeneity (*P* = 0.33, I² = 12%) (Fig. 3C).

##### Sleep efficiency (%)

The pooled analysis for sleep efficiency showed no significant difference between both groups (MD = 1.04% [-1.94, 4.02], *P* = 0.49), with low heterogeneity (*P* = 0.86, I² = 0%) (Fig. 3D).

##### Sleep latency (minutes)

**Fig. 4:**
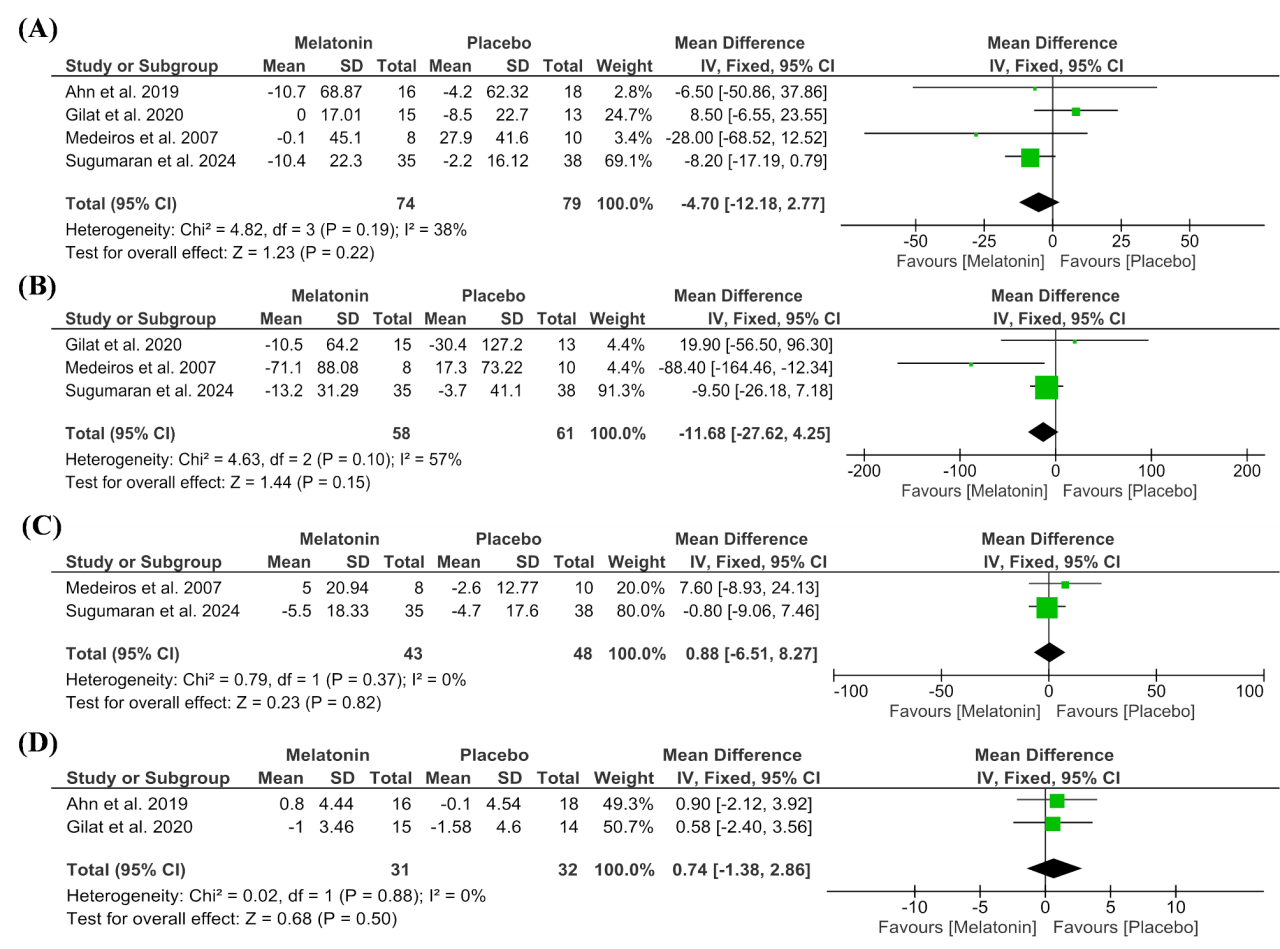
Forest plots of sleep-related outcomes, including (**A**) Sleep Latency (minutes), (**B**) REM Sleep Latency (minutes), (**C**) Frequency of Arousals, and (**D**) REM Sleep Behavior Disorder Screening Questionnaire (RBDSQ Score)

The meta-analysis for sleep latency showed no significant difference between both groups (MD= -4.70 min [-12.18, 2.77], *P* = 0.22), with low heterogeneity (*P* = 0.19, I² = 38%) (Fig. 4A).

##### REM sleep latency (minutes)

Three studies reported the outcome of REM sleep latency. The analysis showed no significant difference between the two groups (MD = -11.68 min [-27.62, 4.25], *P* = 0.15). The pooled studies were moderately heterogeneous (*P* = 0.10, I² = 57%), and heterogeneity was best resolved by excluding Medeiros et al. (MD = -8.16 min [-24.46, 8.13], *P* = 0.33), heterogeneity (*P* = 0.46, I² = 0%) (Fig. 4B).

##### Frequency of arousals

Regarding the frequency of arousals, the pooled studies showed no significant difference between both groups (MD = 0.88 times [-6.51, 8.27], *P* = 0.82), with low heterogeneity (*P* = 0.37, I² = 0%) (Fig. 4C).

##### REM sleep behavior disorder screening questionnaire (RBDSQ Score)

Regarding the RBDSQ Score, the pooled analysis showed no statistically significant difference between the groups (MD = 0.74 [-1.38, 2.86], *P* = 0.50), with low heterogeneity (*P* = 0.88, I² = 0%) (Fig. 4D).

#### Parkinson’s disease-related outcomes

##### UPDRS part III scores

**Fig. 5:**
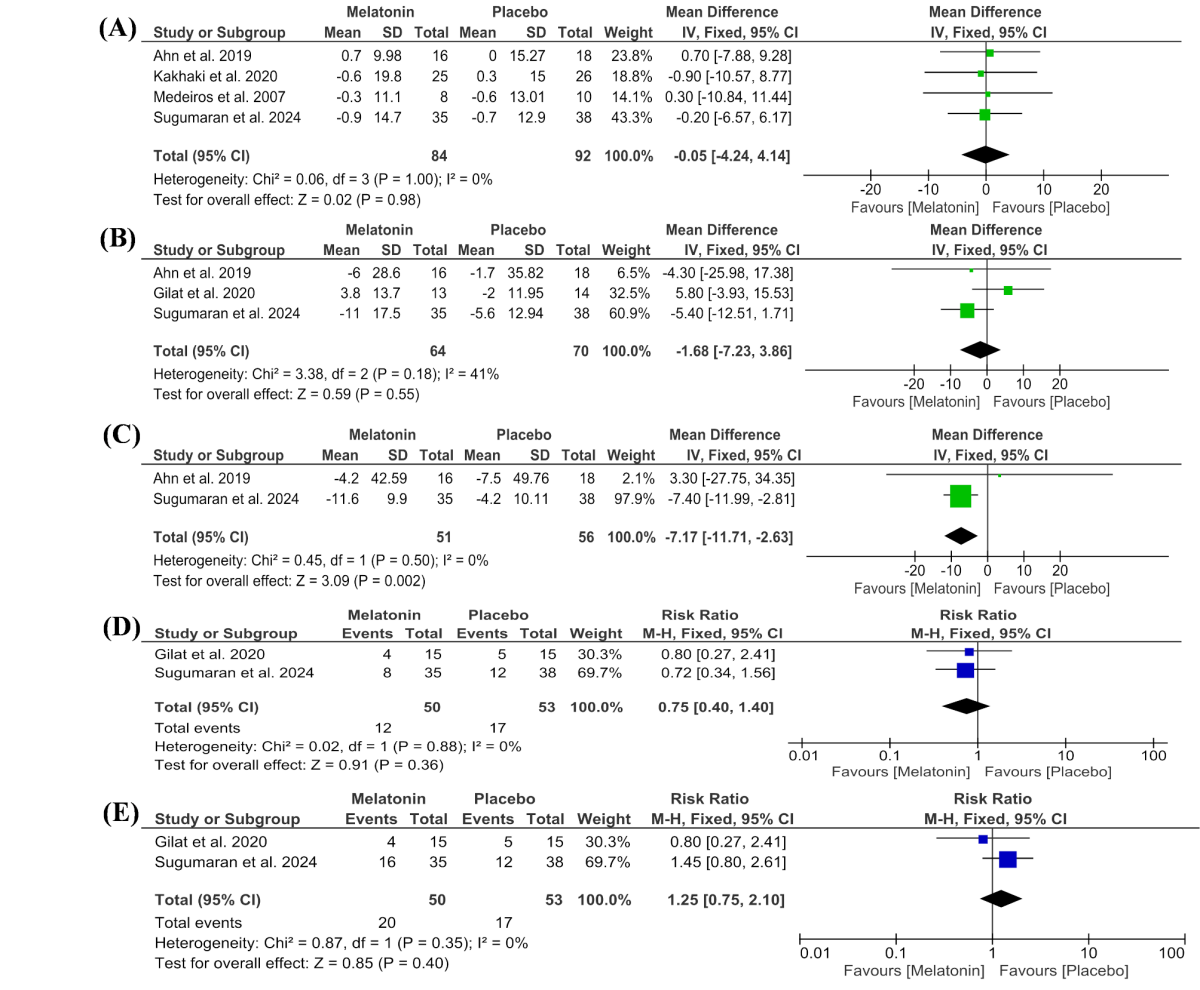
Forest plots of outcomes, including (**A**) UPDRS Part III scores, (**B**) Parkinson’s Disease Quality of Life (PDQ-39), (**C**) Non-Motor Symptom Scale (NMSS), (**D**) Headache, and (**E**) Fatigue

The pooled meta-analysis of the included studies demonstrated no significant improvements in the UPDRS Part III score between the melatonin and placebo groups (MD = -0.05 [-4.24, 4.14], *P* = 0.98), with low heterogeneity (*P* = 1.00, I² = 0%) (Fig. 5A).

##### Parkinson’s disease quality of life (PDQ-39)

The meta-analysis of the PDQ-39 showed no significant improvement in the (MD = -1.68, CI: [-7.23, 3.86], *P* = 0.55), with low heterogeneity (*P* = 0.18, I² = 41%) (Fig. 5B).

##### Non-motor symptom scale (NMSS)

The pooled studies of the NMSS showed a significant difference between both groups (MD= -7.17, CI: [-11.71, -2.63], *P* = 0.002), with low heterogeneity (*P* = 0.50, I² = 0%) (Fig. 5C).

#### Side effects

Two studies reported adverse effects, including headache and fatigue. No significant differences were observed between the melatonin and placebo groups (RR = 0.75, 95% CI: [0.40, 1.40], *P* = 0.36; RR = 1.25, 95% CI: [0.75, 2.10], *P* = 0.40, respectively). Low heterogeneity was noted (I² = 0%) (Fig. 5C and D).

### Certainty of evidence assessment

The GRADE assessment of the findings and the certainty of evidence are presented in Supplementary Material 2. The sleep-related outcomes demonstrated a moderate level of evidence certainty, as did the UPDRS-III outcome. In contrast, the PDQ-39 outcome was of low quality due to imprecision and heterogeneity.

## Discussion

In this meta-analysis, we synthesized evidence from RCTs involving PD patients with sleep disorders to assess the efficacy of melatonin in improving sleep parameters and other PD-related scores. The findings indicate that melatonin significantly improves subjective sleep quality, as measured by the PSQI, suggesting its potential to enhance sleep perception. Additionally, improvements were observed in non-motor symptoms of PD, as assessed by the NMSS. However, no significant differences were found for other sleep-related outcomes, including the ESS, total sleep time, sleep efficiency, sleep latency, REM sleep latency, frequency of arousals, or RBDSQ scores. Similarly, melatonin did not yield significant improvements in motor symptoms (UPDRS Part III scores) or PDQ-39 in PD patients. Regarding the safety of melatonin, the occurrence of adverse events like headache and fatigue showed no significant difference between the melatonin and placebo groups, indicating a favorable safety profile.

Regarding the PSQI —a validated and widely recommended tool for evaluating subjective sleep quality [32]— The statistically significant improvement observed in our study is consistent with findings from previous meta-analyses, Ma et al. [33] which have consistently demonstrated melatonin’s efficacy in improving subjective sleep quality in PD patients. Furthermore, Hadi et al. [34] reported that a 4-week intake of melatonin led to a significant reduction in PSQI scores from baseline compared to other interventions, such as trazodone and clonazepam. The enhancement in PSQI scores highlights melatonin’s potential to improve perceived sleep quality, which is a key concern for individuals struggling with sleep disturbances, as a prominent component of their non-motor symptom profile. While the minimal clinically important difference (MCID) for the PSQI in PD populations has not been definitively established, we used a 3-point reduction, which is commonly used as a conservative estimate. Although the observed improvement in PSQI was statistically significant, it did not meet this commonly accepted threshold, suggesting that the clinical benefit may be modest. Furthermore, the absence of notable improvements in objective sleep metrics, such as total sleep time and sleep efficiency, indicates that melatonin’s effects may be more apparent in subjective perceptions of sleep rather than in objectively measured sleep parameters.

In an 8-week crossover trial, Dowling et al. [17] reported that both 5 mg and 50 mg doses of melatonin were safe, with a statistically significant improvement in total sleep time observed with the 50 mg dose compared to the 5 mg dose or placebo. However, this improvement (10 min) was short and may lack clinical significance. Our meta-analysis corroborates these findings, showing no significant differences in total sleep time between the melatonin and placebo groups.

Daytime sleepiness is a prevalent and impactful non-motor symptom in PD patients, significantly affecting their quality of life [35]. Our analysis of the ESS revealed no significant difference between the melatonin and placebo groups in alleviating daytime sleepiness. This finding aligns with previous studies, including Ma et al. [33] and the crossover trial by Delgado-Lara et al. [36]. Both of them reported persistent abnormal daytime and nighttime sleepiness despite melatonin administration. These results suggest that melatonin may not be effective in addressing daytime sleepiness in PD patients. This highlights the need for alternative therapeutic strategies to manage this debilitating symptom.

RBD affects approximately one-third of PD patients [37]. Beyond disrupting sleep, RBD heightens the risk of cognitive decline and dementia [38] emphasizing the need for early identification and management. Despite its clinical relevance, RBD is frequently overlooked in routine practice [39], necessitating the use of screening tools such as the RBDSQ and gathering detailed history from patients and partners to improve diagnostic accuracy [40]. In their three-arm trial, Hadi et al. reported that melatonin intake was associated with a significant decrease in RBDSQ scores compared to clonazepam (*P* = 0.004) and trazodone (*P* = 0.011) [34]. However, our pooled analysis showed no difference between the melatonin and placebo groups. Furthermore, individual studies reported no improvement in RBDSQ scores with melatonin. This discrepancy may be attributed to the limited number of trials investigating this outcome.

The significant reduction in NMSS scores is a promising finding, suggesting that melatonin may alleviate non-motor symptoms in PD patients. This aligns with emerging evidence highlighting melatonin’s potential neuroprotective and anti-inflammatory properties, which could indirectly improve non-motor symptoms [12, 14]. However, the lack of improvement in UPDRS Part III scores and PDQ-39 suggests that melatonin’s benefits may be limited to specific domains of PD symptomatology. This is consistent with the findings of Liguori et al. [41], who reported no significant improvement in motor dysfunction (UPDRS Part III) following melatonin administration [41]. These results suggest that while melatonin holds potential as an adjunct therapy for non-motor symptoms in PD, its efficacy in addressing motor impairments remains limited.

While melatonin has shown a promising improvement in subjective sleep quality and select non-motor symptoms in PD, its effects on objective sleep parameters, motor function, and quality of life remain limited. Compared to pharmacologic treatments such as clonazepam—a long-acting benzodiazepine considered a first-line option for RBD but often limited by adverse effects like daytime sedation, dizziness, and fall risk—and trazodone, an atypical second-generation antidepressant with relatively favorable tolerability in older adults and complex activity across various pathways [34], melatonin offers a more benign safety profile but a modest therapeutic effect. In parallel, non-pharmacologic strategies, cognitive behavioral therapy for insomnia (CBT-I) has demonstrated benefit in both general and PD populations [42]. Notably, a meta-analysis by Luo et al. (2021) reported that CBT-I produced a moderate improvement in insomnia severity and a mild improvement in sleep quality among PD patients [42]. Given these considerations, future studies should seek to clarify their comparative effectiveness and explore their integration into broader, multimodal management approaches—while controlling for factors such as age-related sleep changes, medication-induced disruption, and comorbidities in order to better clarify melatonin’s role within the broader therapeutic landscape.

### Limitations

Although this meta-analysis employs a comprehensive search strategy and strict inclusion criteria, enhancing its robustness, some limitations should be acknowledged. The low statistical heterogeneity observed across most outcomes (I² < 50%) further strengthens the reliability of the combined estimates. However, the sources of potential clinical heterogeneity include differences in melatonin dosages ranging from 2 to 10 mg, treatment durations (from 4 weeks to 12 weeks), and time since diagnosis of PD. It should be acknowledged that such clinical variability may have influenced the pooled effect estimates and limits the generalizability of the results. Other limitations also warrant consideration. First, the small number of studies for some outcomes, such as REM sleep latency and adverse effects, may limit the generalizability of the findings. Second, the included studies varied in melatonin dosage and follow-up durations, which could have influenced the results. Third, the lack of systematic assessment of patient-reported outcomes (PROs) beyond PSQI and NMSS. Specifically, measures such as sleep satisfaction, sleep diaries, and caregiver burden were not consistently evaluated across the included studies. Sleep diaries can provide valuable insights into patients’ subjective sleep patterns and disturbances, offering a more nuanced understanding of sleep quality over time. Additionally, caregiver burden is a critical aspect to consider, as sleep disturbances in PD patients have been associated with increased caregiver stress and reduced quality of life [43]. Future clinical trials should incorporate these PROs to capture a more comprehensive picture of the impact of interventions like melatonin on both patients and their caregivers.

A Larger, high-quality RCT is needed to confirm the effects of melatonin on objective sleep measures and non-motor symptoms in PD. Second, studies should explore the optimal dosage and timing of melatonin administration to maximize its benefits. Third, research should investigate the long-term effects of melatonin on sleep and PD-related outcomes, as well as its potential interactions with other treatments. There is also a need to address other sleep disorders such as obstructive sleep apnea, periodic leg movements during sleep, and restless leg syndrome.

## Conclusion

In conclusion, this meta-analysis provides evidence that melatonin improves subjective sleep quality and non-motor symptoms in PD patients, with good safety and tolerability. However, its effects on objective sleep measures, motor symptoms, and quality of life remain inconclusive. These findings highlight the need for larger, high-quality RCTs that investigate the long-term effects of melatonin, explore optimal dosing strategies, and assess its role in combination with other pharmacologic or behavioral interventions. Further research is warranted to provide a clearer understanding of melatonin’s role in the therapeutic landscape of PD.

## Electronic Supplementary Material

Below is the link to the electronic supplementary material.

Supplementary Material 1: Search Strategy for each database

Supplementary Material 2: Summary of outcomes and certainty of evidence based on the GRADE approach

## Abbreviations

PD: Parkinson’s Disease
UPDRS: Unified Parkinson’s Disease Rating Scale
PDQ: Parkinson’s Disease Questionnaire
ESS: Epworth Sleepiness Scale
NMSS: Non-Motor Symptoms Scale
RBDQS: REM Sleep Behavior Disorder Screening Questionnaire
RCT: Randomized Controlled Trial
MD: Mean Difference
RR: Risk ratio
CI: Confidence Interval
