# Supplementary material for: Exploring the role of melatonin in managing sleep and motor symptoms in Parkinson’s disease: a pooled analysis of double-blinded randomized controlled trials
Source: Neurol Sci. 2025 May 19;46(9):4155–68. doi: 10.1007/s10072-025-08221-8 (PMC12394315; doi:10.1007/s10072-025-08221-8)
Supplement: Supplementary file 1 — Supplementary Material 1: Search Strategy for each database [file 10072_2025_8221_MOESM1_ESM.docx]

| **Supplementary 1. Search Strategy for each database.** | |  |
| --- | --- | --- |
| **Database** | **Strategy** | **results** |
| **PubMed** | ("melatonin*" OR "5-methoxy-N-acetyltryptamine" OR "melatonine" OR "melatonin receptor agonist*" OR "N-acetyl-5-methoxytryptamine" OR "Circadin" OR "Rozerem" OR "ramelteon" OR "agomelatine" OR "tasimelteon" OR "melatonin agonists" OR "melatonin analog" OR "melatonergic drugs" OR "pineal hormone*" OR "Melatonin"[Mesh]) AND ("Parkinson disease" OR "Parkinson's disease" OR "PD" OR "parkinsonism" OR "idiopathic parkinsonism" OR "primary parkinsonism" OR "Lewy body disease" OR "paralysis agitans" OR "shaking palsy" OR "Idiopathic Parkinson's Disease" OR "Lewy Body Parkinson's Disease" OR "Idiopathic Parkinson Disease" OR "Lewy Body Parkinson Disease" OR "Parkinson Disease"[Mesh]) | 631 |
| **Web of Science** | (ALL=(("melatonin*" OR "5-methoxy-N-acetyltryptamine" OR "melatonin" OR "melatonin receptor agonist*" OR "N-acetyl-5-methoxytryptamine" OR "circadian" OR "rodegem" OR "ramelteon" OR "agomelatine" OR "tasimelteon" OR "melatonin agonists" OR "melatonin analog" OR "melatonergic drugs" OR "pineal hormone*") )) AND ALL=(("Parkinson disease" OR "Parkinson's disease" OR "PD" OR "parkinsonism" OR "idiopathic parkinsonism" OR "primary parkinsonism" OR "Lewy body disease" OR "paralysis agitans" OR "shaking palsy" OR "Idiopathic Parkinson's Disease" OR "Lewy Body Parkinson's Disease" OR "Idiopathic Parkinson Disease" OR "Lewy Body Parkinson Disease") ) | 939 |
| **Scopus** | (TITLE-ABS-KEY(("melatonin*" OR "5-methoxy-N-acetyltryptamine" OR "melatonine" OR "melatonin receptor agonist*" OR "N-acetyl-5-methoxytryptamine" OR "Circadin" OR "Rozerem" OR "ramelteon" OR "agomelatine" OR "tasimelteon" OR "melatonin agonists" OR "melatonin analog" OR "melatonergic drugs" OR "pineal hormone*")) AND TITLE-ABS-KEY(("Parkinson disease" OR "Parkinson's disease" OR "PD" OR "parkinsonism" OR "idiopathic parkinsonism" OR "primary parkinsonism" OR "Lewy body disease" OR "paralysis agitans" OR "shaking palsy" OR "Idiopathic Parkinson's Disease" OR "Lewy Body Parkinson's Disease" OR "Idiopathic Parkinson Disease" OR "Lewy Body Parkinson Disease"))) | 1,823 |
| **Cochrane Library** | ("melatonin*" OR "5-methoxy-N-acetyltryptamine" OR "melatonine" OR "melatonin receptor agonist*" OR "N-acetyl-5-methoxytryptamine" OR "Circadin" OR "Rozerem" OR "ramelteon" OR "agomelatine" OR "tasimelteon" OR "melatonin agonists" OR "melatonin analog" OR "melatonergic drugs" OR "pineal hormone*") in Title Abstract Keyword AND ("Parkinson disease" OR "Parkinson's disease" OR "PD" OR "parkinsonism" OR "idiopathic parkinsonism" OR "primary parkinsonism" OR "Lewy body disease" OR "paralysis agitans" OR "shaking palsy" OR "Idiopathic Parkinson's Disease" OR "Lewy Body Parkinson's Disease" OR "Idiopathic Parkinson Disease" OR "Lewy Body Parkinson Disease") in Title Abstract Keyword | 165 |
| **Total results** |  | 3558 |
